# Deer Antler *δ* ^18^O Records Desalination in Israel's Water Supply

**DOI:** 10.1002/rcm.70139

**Published:** 2026-07-20

**Authors:** Corrin Laposki, Audra Darcy, Noam Werner, Gideon Hartman

**Affiliations:** ^1^ Science Research Initiative, College of Science University of Utah Salt Lake City Utah USA; ^2^ Department of Anthropology University of Connecticut Storrs Connecticut USA; ^3^ The Louis Ariel Goldschmidt Haifa Educational Zoo Haifa Israel; ^4^ The Tisch Family Zoological Gardens in Jerusalem Jerusalem Israel; ^5^ IUCN SSC Deer Specialist Group Gland Switzerland; ^6^ Department of Sociology and Anthropology Ben‐Gurion University of the Negev Beer‐Sheva Israel

**Keywords:** anthropogenic water management, bioapatite carbonate, isoscape‐based provenance, reverse osmosis desalination, stable oxygen isotopes

## Abstract

**Rationale:**

Stable oxygen isotope ratios (*δ*¹⁸O) in mammalian tissues are widely used to reconstruct past climates, seasonal patterns, and animal origins. These analyses assume that animals drink local rainwater—an assumption now challenged by large‐scale anthropogenic water management. Here we present a *δ*¹⁸O time series from Mesopotamian fallow deer ( *Dama mesopotamica* ) antler carbonate spanning 2006–2021, a period coinciding with the major expansion of desalinated water in Israel's national grid.

**Methods:**

Antler samples were collected across 2006–2021, and carbonate *δ*¹⁸O values were measured using an automated carbonate preparation device (KIEL‐III) coupled to a gas ratio mass spectrometer (Finnigan MAT 252) at the University of Arizona's Environmental Isotope Laboratory. Measurements were calibrated against repeated analyses of NBS‐19 and NBS‐18 standards; analytical precision was ±0.1‰ for *δ*¹⁸O (1σ).

**Results:**

Antler *δ*¹⁸O values show a systematic shift toward isotopically elevated compositions through time. This temporal trajectory closely tracks the sequential commissioning of five major reverse osmosis facilities between 2005 and 2015. One‐way ANOVA confirmed significant differences in *δ*¹⁸O across pre‐desalination, transitional, and post‐expansion sample groups (F(2,33) = 11.21, p < 0.001).

**Conclusions:**

These findings demonstrate that anthropogenic water management can induce isotopic shifts in mammalian tissues that rival natural climatic variability, introducing systematic errors into provenance reconstructions. Researchers working with modern or recent historical faunal assemblages in water‐managed landscapes should incorporate documented water supply histories into their interpretive frameworks alongside climatological isoscapes.

## 1 Introduction

The oxygen isotope composition (*δ* ^18^O) of mammalian skeletal tissues is a well‐established proxy for that of consumed water, which in turn reflects local precipitation and hydrologic systems [1, 2, 3]. As meteoric water *δ* ^18^O is reliably patterned across geography and climate, tissue isotope values are routinely used to infer animal provenance and mobility, reconstruct paleoclimate, and interpret subsistence strategies in archaeological contexts [3]. The fidelity of oxygen isotopes derived from mammalian tissues as reliable proxies rests on the assumption that the water consumed approximates local meteoric or groundwater *δ* ^18^O values, which are themselves predictable from precipitation patterns [4, 5]. For wild animals, this assumption holds water—so to speak. Free‐ranging animals encounter plant and drinking water with isotopic values shaped by precipitation, ground water recharge, rivers, lakes, and local geography. However, human population pressure and climate change have resulted in anthropogenic interference in water systems worldwide [6, 7, 8]. Animals within the scope of anthropogenically modified water sources may therefore have tissue *δ* ^18^O values that reflect this interference over that associated with natural water systems.

Stable isotopes have been proposed as key tools for investigating anthropogenic environmental change [9, 10], yet we must equally consider how that change shapes isotopic signals themselves. This is especially critical when constructing isoscapes—spatial models linking geography to stable isotope values in metabolically inert tissues such as keratin and enamel—which underpin isotope‐based forensic and provenance methods [11, 12]. Where animals or plants draw on strongly anthropogenically modified water sources, their tissues may record human intervention rather than the natural environmental signal these isoscapes are designed to capture. Failure to account for this decoupling risks embedding managed water signatures into baselines used to interpret past and present animal and plant dynamics, potentially misattributing anthropogenic effects to climatic or ecological drivers.

**FIGURE 1:**
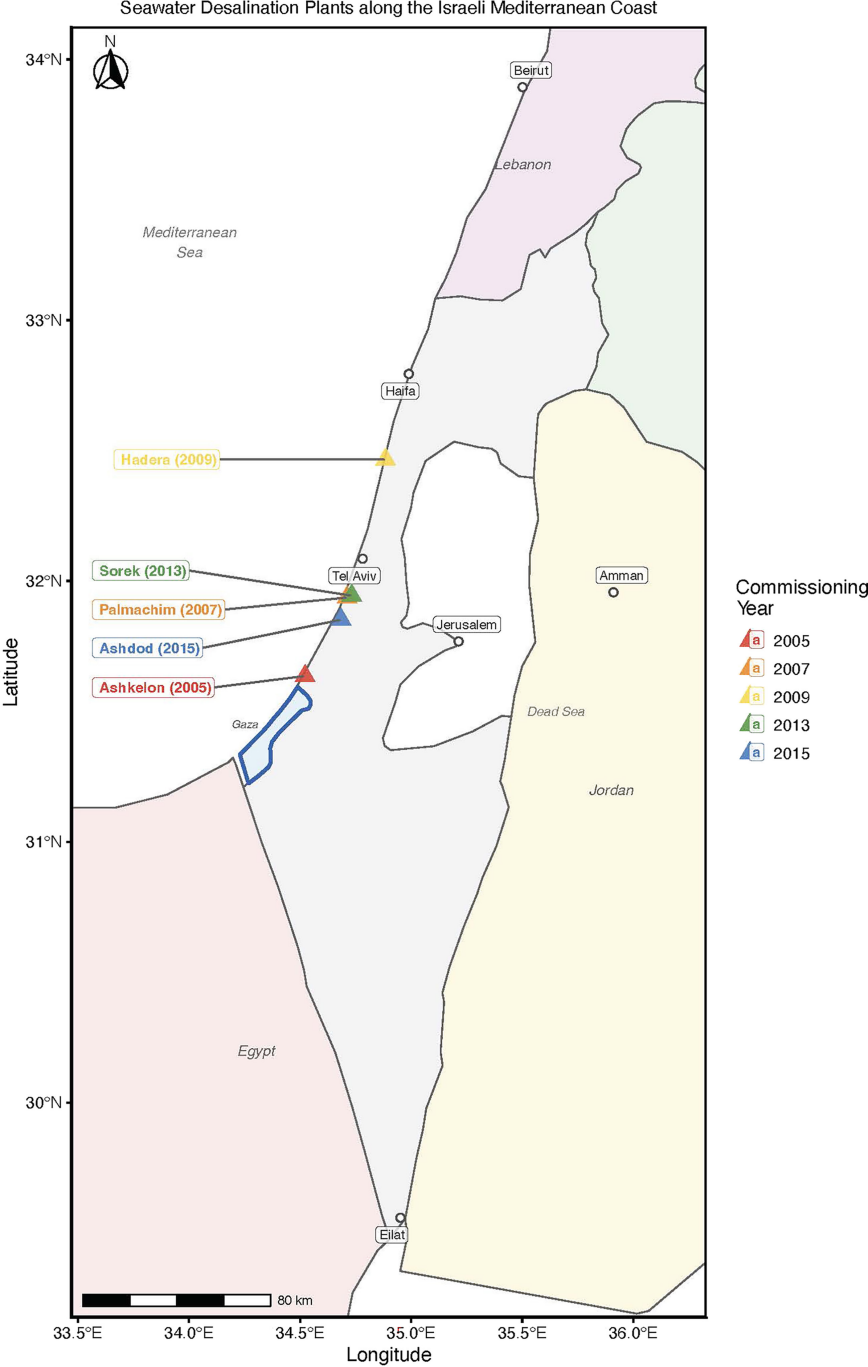
Map of Israel showing the locations of the five major seawater reverse osmosis desalination plants along the Mediterranean coast, color‐coded by commissioning year. Plants were commissioned sequentially between 2005 and 2015: Ashkelon (2005), Palmachim (2007), Hadera (2009), Sorek (2013), and Ashdod (2015). Boundary data from Natural Earth; plant locations verified via Google Maps.

Israel presents an ideal experiment in this regard. Facing chronic water scarcity and regular supply deficits, the Israeli government pursued large‐scale seawater desalination as a national strategy in 1999 [8, 13]. This resulted in the sequential commissioning of five major reverse osmosis (RO) facilities: Ashkelon (2005), Palmachim (2007), Hadera (2009), Sorek (2013), and Ashdod (2015) (Figure 1). By 2020, desalination supplied approximately 50% of Israel's domestic water needs [8], with a sixth plant planned for the north and a second Sorek facility (Sorek 2—Be′er Miriam) opened as of 2025 [14]. Although oxygen isotopes are not fractionated during reverse osmosis [15], seawater has naturally elevated ^18^O values due to preferential evaporation of ^16^O [16]. Desalinated water (DSW) from the Hadera plant has a *δ* ^18^O value of +1.41‰, whereas groundwater in the region ranges from −4.48‰ to −5.43‰ [17]. Consequently, as desalinated seawater became an increasing proportion of the drinking water supply, consumer tissue *δ* ^18^O would be expected to increase accordingly.

Israeli precipitation *δ* ^18^O varies spatially from approximately −1.8‰ to −7.3‰ VSMOW across elevation and aridity gradients [18, 19, 20]. The resulting ~8‰ offset between desalinated and meteoric water represents a resolvable geochemical contrast, one readily preserved in the bioapatite of animals consuming this mixed water supply. Deer antler represents a particularly suitable tissue for this investigation. Unlike tooth enamel, which forms during early life and preserves a fixed developmental record [21], antler is regenerated annually and integrates the isotopic composition of water and diet consumed during the months of growth [22, 23]. A time series of antlers spanning the pre‐ and post‐desalination period in Israel thus offers a direct window into how anthropogenic water management alters the isotopic signals preserved in vertebrate tissues, with each antler providing a temporally discrete snapshot of the water isotope environment in the year of its formation.

Here, we present *δ* ^18^O data from deer antler carbonate collected from a zoo population in Israel across 15 years (2006–2021). This span encompasses both the pre‐desalination baseline period and the post‐expansion period following commissioning of all five major RO plants. We compare these data against the expected meteoric *δ* ^18^O baseline in Jerusalem, water samples from Lake Kinneret (Sea of Galilee), DSW from the Hadera plant, and interpret the observed isotopic trajectory in light of the known desalination infrastructure timeline. Our goal is to demonstrate that anthropogenic water management produces a distinctive shift in mammalian tissue *δ* ^18^O that overlays, and may be misinterpreted as, a climatic or ecological signal and to caution that such shifts risk being embedded into isotopic baselines used to interpret past animal and plant dynamics.

## 2 Materials and Methods

### 2.1 Zoo Population

Antler samples in this study were collected across 2006–2021 from captive Mesopotamian Fallow deer ( *Dama mesopotamica* ) bucks at the Tisch Family Zoological Gardens (Jerusalem, Israel). Animals are assigned unique identification numbers at birth. All deer are kept under standardized feeding conditions and receive a uniform diet of cattle kibble, alfalfa, and bitter vetch. Antlers were collected by zoo staff upon natural shedding by individual bucks in January–February of each year. Samples of approximately 1 g were cut from the base of each antler and shipped to the University of Connecticut for downstream isotopic processing. Antler samples from the 2020 and 2021 seasons were prepared for isotopic analysis and the resulting data combined with archival samples collected from the same population in 2006, 2007, 2008, and 2009 using identical methods, yielding a time series spanning the major expansion of Israel's desalination infrastructure.

### 2.2 Water Supply Network

Deer housed in the Tisch Family Zoological Gardens (Jerusalem Biblical Zoo) obtain drinking water via troughs in the enclosure, which are themselves connected to municipal water supplied via the national grid through Hagihon, Jerusalem's municipal water utility. The “skeleton” of Israel's water supply is the National Water Carrier, which transports water from Lake Kinneret southwards, while also blending various other water inputs along the way [24]. Increasingly, this skeleton has incorporated a growing proportion of DSW into the mix [25].

### 2.3 Antler Sampling and Processing

Carbonate extraction followed the protocol of Balasse et al. [26]. Fifteen milligrams of antler powder was obtained using a Dremel rotary tool, and organic residues were removed by immersion in 2.5% NaOCl for 24 h on a rocker plate, followed by five washes with deionized water; samples were vortexed between washes and centrifuged at 13500 × *g* for 2 min. Possible contaminating carbonates (e.g., soil contamination) were subsequently removed by reaction with 0.1 N acetic acid (pH 3; 100 μL per mg of sample) for 4 h, after which samples were washed three times with deionized water using the same vortex and centrifuge procedure. Treated samples were dried overnight at 50°C in a convection oven, weighed into 0.6‐mL microcentrifuge tubes, and submitted for isotopic analysis.

### 2.4 Isotopic Analysis

Stable carbon and oxygen isotopic compositions of antler carbonate were determined using an automated carbonate preparation device (KIEL‐III) coupled to a gas ratio mass spectrometer (Finnigan MAT 252) at the University of Arizona's Environmental Isotope Laboratory. Powdered samples were reacted with dehydrated phosphoric acid under vacuum at 70°C. Stable carbon and oxygen isotopic compositions were calibrated relative to the VPDB scale using repeated measurements of NBS‐19 and NBS‐18 and precision was ±0.1‰ for *δ* ^18^O and ±0.08‰ for *δ* ^13^C (1σ). The isotope ratios are reported as per mil (‰, ^13^C/^12^C, ^18^O/^16^O).

### 2.5 Statistical Analyses

Statistical analyses were performed in R (Version 4.5.2 [27]). Prior to analysis, six samples were excluded on the basis of anomalously negative *δ* ^18^O values, likely reflecting sampling or measurement error, yielding a final analytical dataset of 36 individuals. A subset of individuals contributed antlers across multiple collection years (n=8); these repeated observations were retained as each antler represents an isotopically independent annual record of water consumption.

**FIGURE 2:**
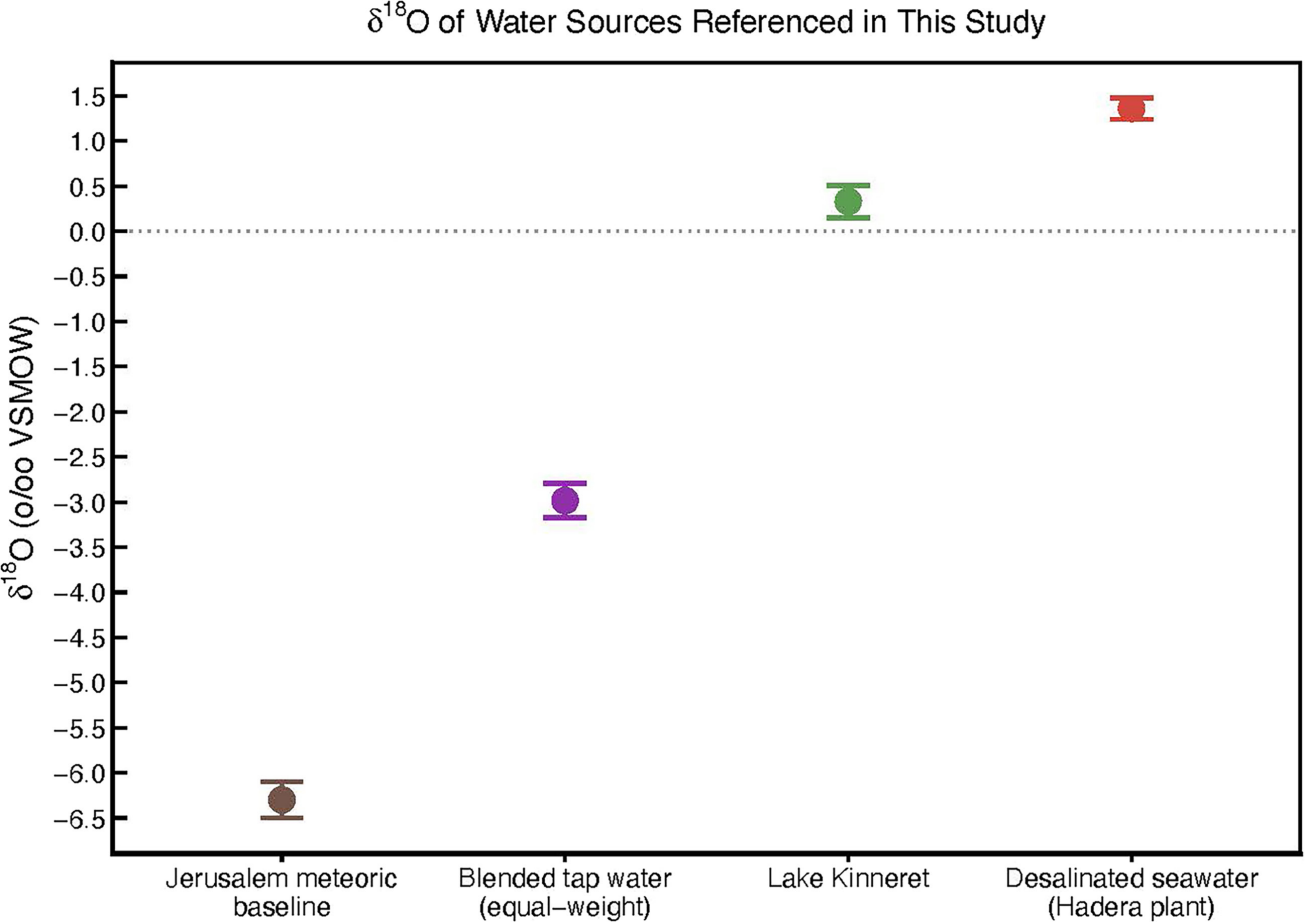
Isotopic composition of water reference end‐members on the VSMOW scale. Points show mean values; error bars show ±1 SD for Lake Kinneret and desalinated seawater, and ±1 SE for the Jerusalem meteoric baseline. The Jerusalem meteoric water baseline (*δ* ^18^O = −6.3‰ ± 0.2‰ SE) is from Goldsmith et al. [19] and Ayalon et al. [18], derived from rainfall sampling spanning the mid‐1990s to early 2000s. Lake Kinneret values (*δ* ^18^O =+0.33±0.18‰) are from Lev et al. [28], sampled in 2009. Desalinated seawater (DSW) from the Hadera plant (*δ* ^18^O = + 1.36‰ ± 0.12‰) is from Ganot et al. [17]. The blended tap water reference (*δ* ^18^O = −2.99‰) represents an equal‐weight mean of the Jerusalem meteoric baseline and Lake Kinneret values; uncertainty is propagated from both sources. Note that antler carbonate *δ* ^18^O values are reported on VPDB and are not directly comparable to these values without scale conversion; VPDB and VSMOW scales are related by $\delta^{18}O_{\text{VSMOW}}=1.03091\times \delta^{18}O_{\text{VPDB}}+30.91$ [29].

Antler carbonate *δ* ^18^O was measured and is reported on the VPDB scale throughout. Water reference values are reported on the VSMOW scale as originally published and are presented for comparison in Figure 2: the Jerusalem meteoric baseline (*δ* ^18^O = −6.3‰ ± 0.2‰ [18, 19]), Lake Kinneret (*δ* ^18^O = +0.33‰ ± 0.18‰ [28])—one of the principal sources of tap water supply to Jerusalem—desalinated seawater (DSW) from the Hadera plant (*δ* ^18^O = +1.36‰ ± 0.12‰ [17]), and an equal‐weight blend of the meteoric baseline and Lake Kinneret values used as an approximate pre‐desalination tap water reference (*δ* ^18^O = −2.99‰).

Samples were assigned to three temporal groups based on the commissioning timeline of desalination plants along the Israeli Mediterranean coast: pre‐desalination (2006), transitional (2007–2009), and post‐expansion (2020–2021). We note that group sizes differ markedly, with the pre‐desalination period represented by only four samples compared to sixteen each in the transitional and post‐expansion groups; this disparity should be borne in mind when interpreting group comparisons. Normality of *δ* ^18^O within each group was confirmed using the Shapiro–Wilk test (pre‐desalination: W=0.894, p=0.402; transitional: W=0.903, p=0.090; post‐expansion: W=0.950, p=0.486). Group differences were assessed using a one‐way analysis of variance (ANOVA) with desalination period as a fixed factor, followed by Tukey's honest significant difference post hoc test for pairwise comparisons. A simple linear regression of *δ* ^18^O against collection year was performed to quantify the directional trend across the full study period. All raw data and R code used to generate the statistics and figures for this study, including a list of the R packages and versions required, are available in the Supporting Information.

### 2.6 AI Disclosure

Portions of this manuscript were prepared with the assistance of Claude Sonnet 4.6 (Anthropic, 2025), a large language model. AI assistance was used for the following tasks: revising grammar and debugging R code for statistical analyses and figure production. All data, interpretations, and conclusions are the sole responsibility of the authors.

## 3 Results

Antler *δ* ^18^O values ranged from −4.19‰ to +1.85‰ VPDB across the full dataset (n=36; Table 1). Descriptive statistics by temporal group are presented in Table 2. Samples from the pre‐desalination period (2006; n=4) yielded a mean *δ* ^18^O of −2.5‰ ± 1.7‰ VPDB, broadly consistent with expected antler carbonate values for animals consuming water with a *δ* ^18^O close to the Jerusalem meteoric baseline of −6.3‰ ± 0.2‰ VSMOW [18, 19] and Lake Kinneret (+0.33‰ ± 0.18‰ VSMOW [28]), after accounting for the known bioapatite–water fractionation offset (α=1.0263±0.0014 [30]). One pre‐desalination sample (D52; *δ* ^18^O = −0.12‰ VPDB) was a notable high outlier relative to the other three pre‐desalination samples, slightly elevating the group mean; although its inclusion does not alter the directionality of the observed trend. The blended pre‐desalination tap water reference, calculated as an equal‐weight mean of these two sources (*δ* ^18^O = −2.99‰ VSMOW), is consistent with the observed pre‐desalination antler values. Transitional period samples (2007–2009; n=16) yielded an intermediate mean of −1.31‰ ± 2.15‰ VPDB, with notably higher variance than either the pre‐desalination or post‐expansion groups. This is to be expected given the progressive shift in municipal water isotopic composition as desalination capacity expanded incrementally during this period. Post‐expansion samples (2020–2021; n=16) showed uniformly positive *δ* ^18^O values with a mean of +0.83‰ ± 0.47‰ VPDB—the tightest distribution of any group—approaching the isotopic composition of desalinated seawater from the Hadera plant (+1.36‰ ± 0.12‰ VSMOW [17]) after bioapatite–water fractionation is accounted for.

**TABLE 1:** Deer antler carbonate *δ* ^18^O values by sampling period.

ID	Year	*δ* ^18^O	ID	Year	*δ* ^18^O	ID	Year	*δ* ^18^O
(‰ VPDB)			(‰ VPDB)			(‰ VPDB)		
Pre‐desalination								
D52	2006	−0.12	D64	2006	−2.77	D92	2006	−4.07
D55	2006	−3.19						
Transitional								
D62	2007	+1.82	D90	2007	−3.23	D16	2009	+1.85
D63	2007	+0.84	D91	2007	−0.71	D76	2009	−0.69
D64	2007	−1.96	D94	2007	−2.97	D83	2009	+1.23
D72	2007	−4.19	D76	2008	−2.79	D92	2009	+0.36
D76	2007	−3.10	D95	2008	−3.77			
D86	2007	−3.81						
D89	2007	+0.21						
Post‐expansion								
D10	2020	+0.10	D70	2020	+0.23	D29	2021	+0.89
D29	2020	+1.03	D10	2021	+1.62	D30	2021	+0.60
D30	2020	+0.66	D11	2021	+1.00	D33	2021	+0.73
D58	2020	+0.87	D112	2021	+1.43	D58	2021	+1.46
D6	2020	+0.26				D60	2021	+0.58
D60	2020	+0.43				D80	2021	+1.44

**TABLE 2:** Descriptive statistics for deer antler carbonate *δ* ^18^O (‰ VPDB) by desalination period.

Group	*n*	Mean δ^18^O	SD	Median δ^18^O	Min	Max
Pre‐desalination (2006)	4	−2.5	1.7	−2.98	−4.07	−0.12
Transitional (2007–2009)	16	−1.31	2.15	−1.33	−4.19	+1.85
Post‐expansion (2020–2021)	16	+0.83	0.47	+0.80	+0.10	+1.62

*Note:* All values on VPDB scale. Shapiro–Wilk: All groups *p >* 0.05. ANOVA: *F*(2,33) = 11.21, *p <* 0.001. Tukey HSD: Post versus pre *p* = 0.002; post versus transitional *p* = 0.001; transitional versus pre *p* = 0.352. Linear regression: *β* = 0.190 (SE = 0.038), *R* ^2^ = 0.42, *F*(1,34) = 24.53, *p <* 0.001.

**FIGURE 3:**
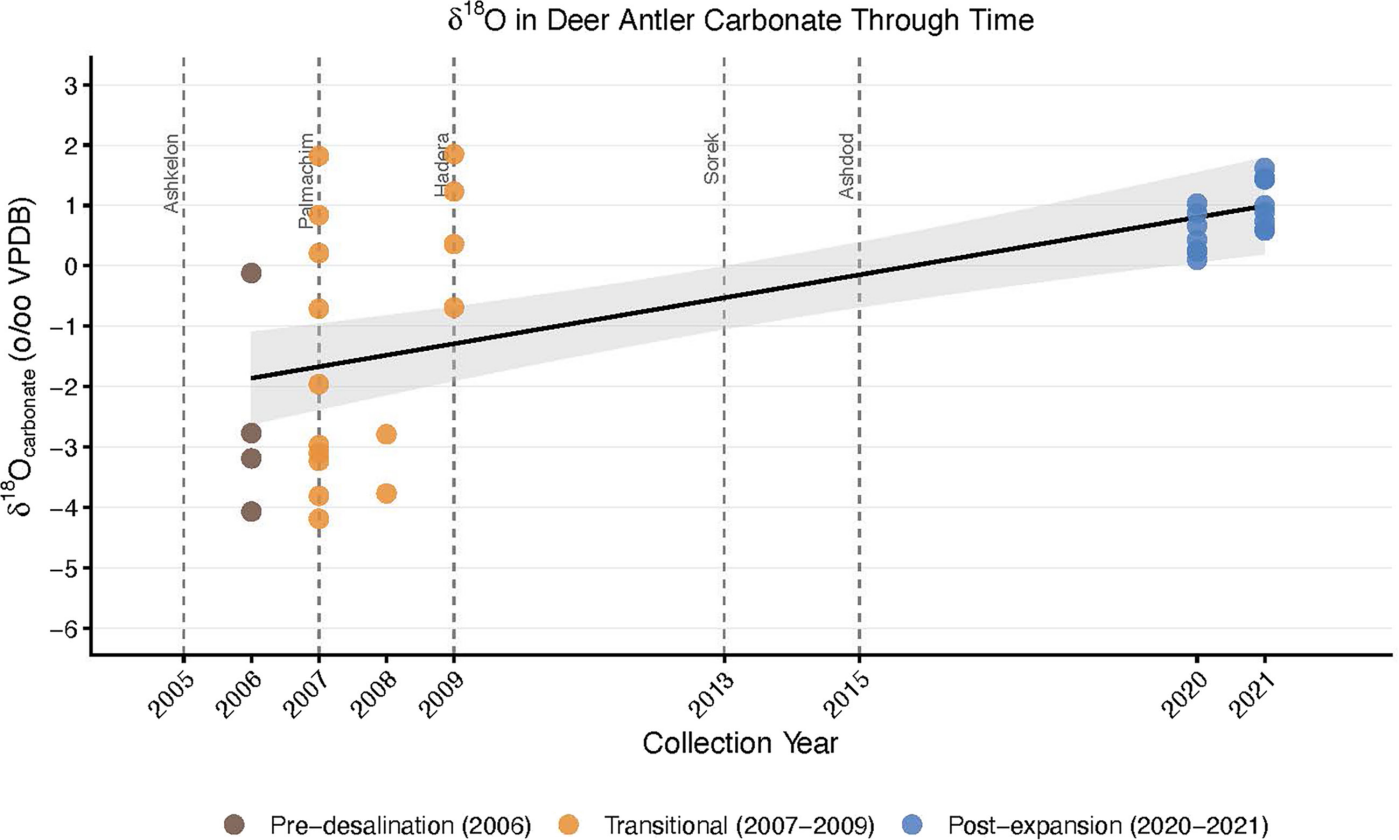
Distribution of antler carbonate *δ* ^18^O (VPDB) in Mesopotamian fallow deer ( *Dama mesopotamica* ) by desalination period. Boxes show the interquartile range with median line; whiskers extend to 1.5 times the interquartile range. Individual observations are shown as jittered points. Group means and standard deviations: pre‐desalination −2.54‰±1.70‰ (n=4); transitional −1.31‰±2.15‰ (n=16); post‐expansion 0.83‰±0.47‰ (n=16). One‐way ANOVA: $F\left(2,33\right)=11.21$, p<0.001. Tukey HSD: post‐expansion versus pre‐desalination p=0.002; post‐expansion versus transitional p=0.001; transitional versus pre‐desalination p=0.352.

Antler carbonate *δ* ^18^O values differed significantly across desalination periods (one‐way ANOVA: $F\left(2,33\right)=11.21$, p<0.001; Figure 3). Post hoc comparisons revealed that post‐expansion values (2020–2021) were elevated relative to both the pre‐desalination period (Tukey HSD: Δ=+3.37, p=0.002) and the transitional period (Tukey HSD: Δ=+2.14, p=0.001), although there was no difference between the pre‐desalination and transitional periods (Δ=+1.23, p=0.352). Significant differences in antler *δ* ^18^O were therefore driven primarily by the contrast between post‐expansion values and both earlier periods.

**FIGURE 4:**
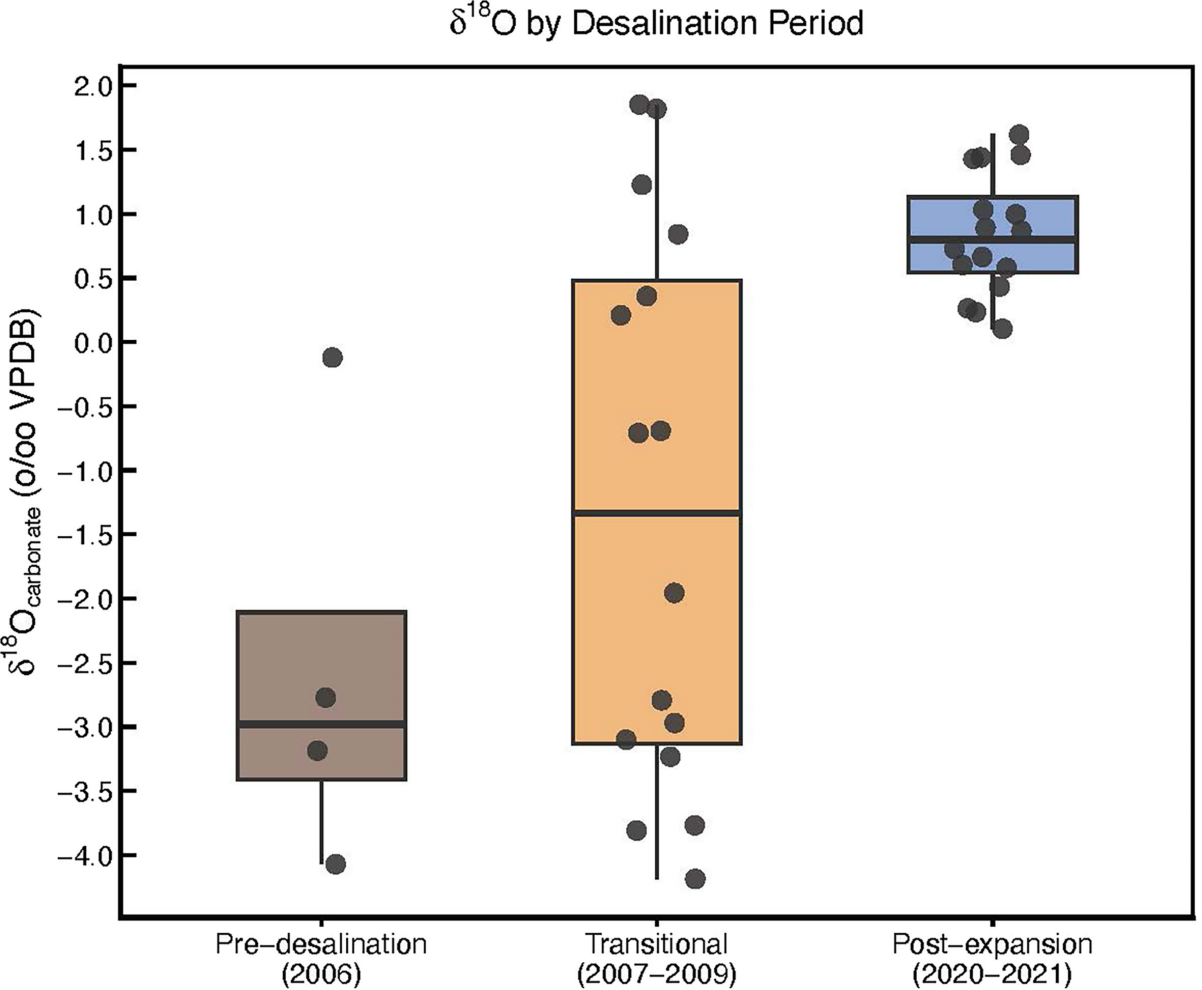
Antler carbonate *δ* ^18^O (VPDB) in Mesopotamian fallow deer ( *Dama mesopotamica* ) through time. Points are colored by desalination period: pre‐desalination (2006, brown), transitional (2007–2009, orange), and post‐expansion (2020–2021, blue). Dashed vertical lines indicate the commissioning years of desalination plants along the Israeli Mediterranean coast (Ashkelon 2005, Palmachim 2007, Hadera 2009, Sorek 2013, and Ashdod 2015). The solid line and shaded band show the ordinary least‐squares regression fit with 95% confidence interval (β=0.190, SE=0.038, $R^{2}=0.42$, $F\left(1,34\right)=24.53$, p<0.001).

A positive linear trend was observed across the full sampling window (2006–2021), with *δ* ^18^O increasing at approximately +0.19‰ per year (β=0.190±0.038; $t\left(34\right)=4.95$, p<0.001; $R^{2}=0.42$; Figure 4). This is consistent with a progressive enrichment of the local water supply following the sequential opening of desalination infrastructure.

## 4 Discussion

### 4.1 Isotopic Decoupling

The shift in antler *δ* ^18^O observed between the pre‐desalination (2006; $\overset{⃐}{x}=-2.54‰\pm 1.70‰$) and post‐expansion (2020–2021; $\overset{⃐}{x}=+0.83‰\pm 0.47‰$) sample groups is difficult to attribute to natural climatic or hydrological drivers. The Jerusalem meteoric water baseline of −6.3‰ ± 0.2‰ VSMOW [18, 19], derived from rainfall sampling spanning the mid‐1990s to early 2000s, indicates no systematic long‐term enrichment trend; the inter‐annual variability (±0.2‰ SE) is far smaller than the +3.4‰ shift observed between our pre‐desalination and post‐expansion groups and cannot account for the observed enrichment. Lake Kinneret, one of the principal pre‐desalination sources of Jerusalem's municipal water supply, has a mean *δ* ^18^O of +0.33 ± 0.18 VSMOW [28], and the equal‐weight blend of these two sources yields a pre‐desalination tap water reference of −2.99‰ VSMOW, akin to the pre‐desalination antler values. A temperature‐driven explanation for the post‐expansion enrichment would require implausibly large regional warming: applying the global meteoric *δ* ^18^O–temperature relationship of 0.69°C^−1^ [31], the observed +3.4‰ shift would imply approximately 5°C of regional warming over the 15‐year study period, inconsistent with observed temperature trends in Israel. Evaporative enrichment of surface water sources would be expected to produce greater seasonal scatter rather than the coherent, low‐variance signal observed in the post‐expansion group (σ=0.47). The most parsimonious explanation is the progressive introduction of isotopically enriched desalinated seawater into Jerusalem's municipal supply. DSW produced by reverse osmosis from Eastern Mediterranean seawater carries a *δ* ^18^O of +1.36‰ ± 0.12‰ VSMOW [17]. This water enters the National Water Carrier directly at coastal pumping stations, where it blends continuously with Kinneret surface water and groundwater inputs, with natural sources adjusted seasonally to meet demand [24]. Israel has interconnected and integrated national and regional supply systems that address the severe disparity in the country's regional water availability. Source integration along the National Water Carrier is geographically asymmetric by design, as arid southern rainfall patterns are 90%–95% lower than those in the temperate north [25]; thus, more southerly regions receive drinking water with a higher proportion of DSW. The convergence of post‐expansion antler values toward the isotopic composition of this desalinated end‐member thus provides the most parsimonious explanation for the observed enrichment, consistent with animals consuming water from an anthropogenically modified supply in which the meteoric signal has been progressively displaced by Mediterranean seawater.

The temporal structure of the shift points clearly to desalination as the primary driver. Our earliest samples (2006) predate large‐scale RO output, and their *δ* ^18^O values align with consumption of meteoric and Lake Kinneret sources. The transitional period (2007–2009) shows greater variance (σ = 2.15‰), likely due to the progressive and uneven introduction of DSW into the national grid as Ashkelon expanded capacity and Palmachim came online. By 2020–2021, following the commissioning of all five major plants and with desalination supplying approximately 50% of domestic water needs [8], antler *δ* ^18^O values had converged on a tight, elevated cluster approaching the Hadera DSW values. The reduction in variance between the transitional and post‐expansion periods is itself informative as it reflects the homogenization of the water supply as desalinated input became a dominant source.

We acknowledge a substantial temporal gap between our transitional (2007–2009) and post‐expansion (2020–2021) sampling windows, coinciding with the commissioning of the Sorek (2013) and Ashdod (2015) facilities and the progressive increase in desalination's share of domestic supply. Whether the isotopic shift occurred gradually through this interval or as a more pronounced step‐change following the commissioning of the larger Sorek and Ashdod plants cannot be resolved from the present dataset. Samples from archival collections spanning 2010–2019, if recoverable, would be particularly valuable for characterizing the trajectory of change and evaluating whether the progressive infrastructure expansion produced correspondingly incremental isotopic shifts in consumer tissues.

RO desalination preserves the isotopic composition of source seawater [15]. When mixed into Israel's National Water Carrier alongside meteoric‐derived and other sources, the resulting mixture is isotopically elevated relative to pre‐desalination inputs. Mesopotamian fallow deer bucks consuming this mixed supply therefore incorporate higher ^18^O into their annually forming antler carbonate. The captive setting of the Tisch Family Zoological Gardens is particularly informative in this regard: The controlled husbandry conditions isolate the water source signal with unusual clarity. This fidelity is consistent with the established physiological basis of mammalian bioapatite oxygen isotope systematics. *δ* ^18^O in body water—and by extension in bioapatite—is governed by the relative contributions of ingested drinking water, dietary water, oxygen bound in food, and inhaled O_2_, each weighted by its flux and fractionation factor [30, 32, 33]. In the captive setting of our study, dietary composition, food‐bound oxygen, and atmospheric O_2_ are likely to have remained relatively constant across the study period. Under these conditions, antler carbonate provides an honest record of environmental water values [34], leaving drinking water *change* as the primary variable governing inter‐annual variation in tissue *δ* ^18^O, though the relative contribution of each oxygen source to bioapatite carbonate was not formally partitioned in this study. This has important implications beyond the present study: where dietary and respiratory oxygen inputs can be assumed stable, bioapatite carbonate *δ* ^18^O serves as a robust proxy for the isotopic composition of local water, whether that water reflects climatic, geographic, or—as demonstrated here—anthropogenic controls on the hydrological cycle.

### 4.2 Implications for *δ* ^18^O as a Paleoenvironmental and Provenance Proxy

Our findings carry considerable methodological implications for isotope‐based zoological and ecological studies conducted in regions with major water management infrastructure. The standard interpretive assumption—that animal tissue *δ* ^18^O tracks local meteoric water—is demonstrably violated when non‐meteoric sources constitute a substantial fraction of the consumed water supply [35, 36]. In Israel's case, the isotopic perturbation is not subtle: The ~8‰ contrast between RO‐desalinated and meteoric water equals or exceeds the full within‐season range of precipitation *δ* ^18^O at Jerusalem, which spans approximately −11‰ to −2‰ across individual events [18], and represents a directional shift that cannot be explained by natural seasonal variability. An investigator applying standard isoscape‐based provenance methods to modern Israeli deer without accounting for desalination would likely misattribute the enriched *δ* ^18^O values to a warmer, more southerly, or more arid region of origin. This problem is not unique to Israel: As large‐scale desalination expands across water‐scarce regions of the world, similar isotopic perturbations may be expected wherever DSW enters open systems accessible to wildlife or agricultural units [37, 38, 39].

Furthermore, as of March 2026, Israel has begun discharging DSW directly into Lake Kinneret via the “Reverse Conveyor” infrastructure project, at a cumulative rate of approximately 5000 cubic meters per hour [40]. This initiative—established under Government Resolution 3866—was inaugurated in response to declining precipitation and climate‐driven reductions in natural inflow [40]. Outside of its status as a national reservoir, Lake Kinneret is a primary freshwater body sustaining its own ecosystem and, via the “Reverse Conveyor” infrastructure project, that of the Tzalmon stream [25]. The accessibility of RO‐altered water to Israeli wildlife is therefore not merely a theoretical concern. Indeed, during the permitting process for the Reverse Conveyor project, Israeli policymakers explicitly weighed the trade‐offs of DSW entering natural stream systems, ultimately concluding that wildlife survival in an increasingly arid landscape depended on access to desalinated sources [25].

For Lake Kinneret and the Tzalmon stream, the isotopic consequences for downstream consumers, including wildlife, livestock, plant life, and human populations, remain to be characterized. However, the progressive replacement of meteoric and surface water sources with isotopically elevated DSW is likely to further complicate isoscape‐based provenance inference across the region and carries other ecological risks, as the DSW has different pH, nutrients, and salinity [41]. Moreover, DSW carries isotopic consequences across multiple trophic levels, as enriched values are propagated from primary consumers to secondary consumers—for example, from dairy cattle consuming DSW [42] to humans consuming their milk. The issue is likely to intensify as climate change drives further expansion of desalination infrastructure globally.

Beyond desalination, our results contribute to evidence that anthropogenic hydrological modification—including inter‐basin water transfer, irrigation return flows, and treated wastewater reuse—can produce systematic biases in mammalian *δ* ^18^O records [43, 44]. As the reach of water infrastructure expands, the assumption of a simple meteoric–tissue isotope linkage requires verification against documented water supply histories rather than climatological predictions alone. This has especially urgent consequences for the forensic use of human tissues—such as tooth enamel or hair—in areas where DSW constitutes a substantial drinking water source, as any local water oxygen and hydrogen isotope values would be overridden by those associated with DSW [45]. We urge researchers working with modern or recent historical faunal assemblages to incorporate documented water supply histories into their interpretive frameworks alongside climatological isoscapes.

### 4.3 Antler as a Biological Monitor of Anthropogenic Water Supply Change

Our results also point to a positive application of this phenomenon. Where the water supply history is well documented, annually regenerated tissues such as antler can serve as biological archives of anthropogenic interference. The annual cycle of antler growth makes it an excellent recorder of the water isotope environment experienced during each growing season. In regions with dateable infrastructure transitions, antler collections held in wildlife management archives, hunting registries, or zoological institution records—such as those utilized here—could provide retrospective timelines of water supply change for periods predating systematic isotopic surveillance. The captive population studied here is particularly well suited to this role given the detailed individual records maintained by the Tisch Family Zoological Gardens.

## 5 Conclusions

We report a temporal shift in Mesopotamian fallow deer ( *D. mesopotamica* ) antler carbonate *δ* ^18^O from the Tisch Family Zoological Gardens, Jerusalem, that tracks closely the sequential expansion of reverse osmosis desalination infrastructure in Israel between 2005 and 2020. Our results show that anthropogenic water management can generate large, directional *δ* ^18^O shifts in vertebrate tissues that are not attributable to climate or ecology. Standard meteoric water‐based interpretive frameworks for tissue *δ* ^18^O may therefore be misleading in water‐managed landscapes, particularly in arid and semiarid regions where desalination infrastructure is rapidly expanding.

## Author Contributions

**Noam Werner:** resources, investigation, data curation. **Corrin Laposki:** conceptualization, investigation, writing – original draft, methodology, visualization, writing – review and editing, formal analysis, data curation. **Gideon Hartman:** conceptualization, investigation, writing – review and editing, methodology, supervision, resources, project administration, funding acquisition. **Audra Darcy:** conceptualization, writing – review and editing.

## Funding

Funding was provided out of Gideon Hartman's start‐up package from the University of Connecticut.

## Conflicts of Interest

The authors declare no conflicts of interest.

## Supporting information


**Data S1:** Supporting Information.

## Data Availability

Raw data and R code used to generate the statistics and figures described in the text are provided as an attached text file.
